# Olfactory training and metacognitive aspects of olfaction in children aged 6–9 years: a preliminary study

**DOI:** 10.1007/s00426-025-02127-y

**Published:** 2025-05-20

**Authors:** Anna Oleszkiewicz, Marta Rokosz, Lukasz Gargula, Daniel Marek, Paulina Nawrocka, Aleksandra Reichert, Kornelia Zienkiewicz, Barbara Zyzelewicz, Agnieszka Sorokowska

**Affiliations:** 1 Department of Otorhinolaryngology, Faculty of Medicine Carl Gustav Carus, Smell & Taste Clinic, Technische Universität Dresden, Dresden, Germany; 2 Institute of Psychology, University of Wroclaw, Wroclaw, 50-527 Poland

## Abstract

People differ in terms of olfactory abilities and awareness of ambient odors. Olfactory training (OT), a method primarily devoted to the rehabilitation of the sense of smell, has been found to have a positive effect on various olfactory and cognitive abilities. Here, addressing an important gap in the literature, we tested experimentally whether odor awareness and significance in young children can be enhanced through standardized OT. Additionally, we explored whether OT can improve odor identification skills and verbal fluency. A sample of 101 children (52 girls) aged between 6 and 9 years (*M* = 7.62 ± 0.61) took part in a 12-week OT, preceded and followed by psychophysical and questionnaire testing. Of these, 57 children were assigned to the experimental group who bi-daily smelled four odors (rose, eucalyptus, lemon and cloves), and 44 children to the placebo group who received odorless samples. Odor significance and awareness score slightly increased, and this improvement was more pronounced in the experimental group. The effect was present in the group that presented lower odor awareness at baseline, suggesting that OT may be particularly effective in children who have lesser experience with odors. OT affected neither the odor identification nor the verbal fluency. We conclude that in children, especially those who have less experience with odors, OT procedure might have certain effects on metacognitive aspects of olfaction. More studies are needed to assess the exact effects of OT on metacognitive olfactory development.

**Supplementary Information:**

The online version contains supplementary material available at 10.1007/s00426-025-02127-y.

## Introduction

Everyday life observations and laboratory studies indicate that people differ in terms of olfactory abilities and awareness of ambient odors. These differences can be physiological, such as the threshold for detecting smells, and psychological, such as the importance of odors and conscious perception of odors in the environment. Some individuals readily notice faint odors and easily recognize smells surrounding them, while others barely detect strong and salient olfactory cues (Croy et al., 2010). Interestingly, studies indicate that olfactory perceptual abilities can be developed through a simple, targeted intervention, i.e., an olfactory training (Hummel et al., 2009; Pieniak et al., 2022; Sorokowska et al., 2017).

The most common olfactory training (OT) procedure involves exposure to four odorants twice a day for 12 weeks (Hummel et al., 2009). The effectiveness of this method was found to be remarkable, particularly in people with olfactory deficits. Simplicity, positive reception among patients, low cost, and absence of any harmful side effects are additional strengths of the olfactory training (Hummel et al., 2009; Pieniak et al., 2022; Sorokowska et al., 2017). A meta-analysis of studies using OT showed its significant, positive effect on the overall olfactory functioning of patients of different smell dysfunction etiologies, with a particularly high effectiveness reported for odor discrimination and identification skills, and slightly lower effects observed in terms of olfactory thresholds (Sorokowska et al., 2017). Although the research outcomes on OT in normosmic participants are less straightforward [for a review see (Pieniak et al., 2022)], olfactory training was found to positively affect odor detection abilities (Oleszkiewicz et al., 2022), and to prevent olfactory deterioration (Schriever et al., 2014). The effects of standardized training are in line with the enhanced olfactory performance in professionals, functioning daily in odor-focused disciplines, such as sommeliers, perfumers, and beer experts (Bende & Nordin, 1997; Parr et al., 2002; Zucco et al., 2011).

The positive effects of the OT can be observed also on a central nervous system level (Pieniak et al., 2022), as it may increase olfactory bulb volume (Negoias et al., 2017) and cortical thickness in olfactory perception-related areas (Al Aïn et al., 2019). The neuroanatomical effects of olfactory training open novel and fascinating research questions on its potential relationship with areas of human functioning other than sensory perception. Olfactory processing areas are interrelated with the limbic system, suggesting some potential effects of olfactory training on emotions, learning, and memory. This line of thinking has so far been explored in just a handful of studies. For example, verbal fluency and subjective well-being improved after olfactory training, but not after the “sudoku task training” in elderly people (Birte-Antina et al., 2018). A significant improvement in verbal fluency following olfactory training has also been shown in normosmic and dysosmic adults subject to olfactory training (Oleszkiewicz et al., 2022), although this effect did not generalize to phonetic verbal fluency (Oleszkiewicz et al., 2021a, b, 2022). The beneficial, cognitive effects of OT again find support on a neuroanatomical level. Following olfactory training, patients with post-traumatic olfactory loss exhibited significantly increased activation in semantic processing brain areas (Pellegrino et al., 2019).

Despite the rapid expansion of research on OT (Pieniak et al., 2022), and some studies on adults in the relatively unexplored area of cognitive and emotional effects of OT, not much is known about the olfactory training in children. Provided the dynamic cognitive and emotional, but also olfactory development in young people, this seems to be a very interesting literature gap to address. The results of the three available reports involving healthy children (Mahmut et al., 2021a, b; Mori et al., 2015) are positive, yet not fully consistent. Mori and colleagues (Mori et al., 2015) showed an improvement in thresholds for odor detection in a group of 9- to 14-year-old children after OT, and Mahmut and colleagues (Mahmut et al., 2021a, b) reported an improvement in odor identification, but not in odor detection, in a group of 8-year-olds. Olfactory training involving odors of low concentration increased olfactory sensitivity also in children after mild traumatic brain injury, although this was observed only for odor detection, and not for odor identification tasks. Additionally, olfactory perception in the healthy control group in that study remained unchanged (Pieniak et al., 2023). Even less is known about OT effects in areas of children’s functioning beyond olfaction. Exposure to odors was found to have a positive effect on olfactory function and pain threshold in children and adolescents with primary headaches (Gossrau et al., 2023), and similar, positive outcomes of OT with odors of low concentration were also observed for fluid intelligence in children after mild traumatic brain injury as well as healthy controls (Pieniak et al., 2023). Apart from these clinical reports, however, little can be said on emotion- or cognition-related changes in children following olfactory training.

In addition to the more “objective”, or psychophysical aspects of olfactory perception, people differ in terms of their focus on and awareness of the olfactory world. Olfactory cues can drive, affect and influence emotions and behaviors of some individuals, leaving others completely unaffected. The level of attention paid to odors and the degree of their importance in everyday functioning appears in the literature as “significance of olfaction” (Croy et al., 2010; Lohrer et al., 2022), “importance of olfaction” (Sorokowski et al., 2023), “odor awareness” (Smeets et al., 2008), or “odor reactivity and awareness” (Ferdenzi et al., 2008a, b; Saxton et al., 2014) and can be measured using several existing scales, slightly differing in terms of targeted areas of functioning and studied groups (Croy et al., 2010; Ferdenzi et al., 2008a, b; Lohrer et al., 2022; Smeets et al., 2008; Sorokowski et al., 2023). Despite using different labels, researchers agree that this metacognitive construct captures a considerable inter-individual variation in the importance of olfactory information in various aspects of daily smell-related functioning (Martinec Nováková & Havlíček, 2019). The self-reports on olfactory perception provide somewhat divergent information from regular olfactory testing, and may vary regardless of the actual olfactory acuity (Ferdenzi et al., 2010; Sorokowska et al., 2019). The level of attention paid to odors may drive the potential search for pleasant olfactory stimuli in commercial decisions (Croy et al., 2010), moderate the importance of odors in social situations, or influence emotional responses to odors and resulting behaviors (Ferdenzi et al., 2008a, b).

Interestingly, intense focus on daily olfactory perception may lead to an improvement in olfactory acuity (Oleszkiewicz et al., 2021a, b), empirically supporting the notion that odor awareness relies on associative learning processes (Dalton & Hummel, 2000). Hence, odor awareness and olfactory acuity may interact with each other, and it can be hypothesized that OT can affect odor awareness. However, there are very few studies in this area, and even less is known about such potential effects in children. It seems that olfactory awareness and odor significance may change with age, likely mirroring the changes in the general olfactory development (Ferdenzi et al., 2008a, b;, Martinec Nováková & Havlíček, 2019; Saxton et al., 2014). A particular age-related increase was observed, for example, in food- and environment-related olfactory perception (Martinec Nováková & Havlíček, 2019) [but see (Martinec Nováková & Vojtušová Mrzílková, 2016; Nováková et al., 2018)]. The only existing semi-longitudinal study targeting children’s odor awareness investigated the effects of various proxies of olfactory experience (parental self-reports of odor awareness and parental reports of the children’s olfaction-related activities) on children’s odor awareness over the period of one and a half years (Nováková et al., 2018). The children whose parents reported that they had greater experience with diverse food-related odors were reported to exhibit higher odor awareness/olfactory reactivity scores. Nevertheless, it would be beneficial to expand the knowledge in this area, particularly using the standardized olfactory training. For example, young schoolchildren are likely to benefit from OT provided the intense development of their memory (Gathercole, 1998) and verbal skills (Gathercole et al., 1992) at this stage of their lives. They also present coherent, growing olfactory abilities (Gellrich, Stetzler, Oleszkiewicz, Hummel, & Schriever, 2017; Schriever et al., 2018a, b). Therefore, in the current study, we intended to explore whether odor awareness and significance of olfaction may be developed through olfactory training in young children. In the study protocol, we additionally included olfactory identification ability and verbal fluency, as it is known to modulate children’s odor awareness (Ferdenzi et al., 2008a, b). This study was also motivated by its potential practical implications. The educational program for primary schools marginally tackles sensory hygiene with almost no attention paid to the sense of smell. With this study, we are hoping to deliver practical tools for the teachers and educators to apply in the classroom to raise awareness about olfaction in young children. Furthermore, demonstrating the metacognitive effects of olfactory training may find application in interventions intended to shape healthy eating habits. Smell is an important sense guiding food choice, and European societies are facing an epidemic of childhood obesity (Murray et al., 2020). Thus, the search for new methods to support children in daily healthy habits is a pressing problem, and this study aimed to pave a path towards solving it.

## Methods

### Compliance with ethical standards

This study was conducted in line with the principles of the Declaration of Helsinki. Ethical approval was received from the institutional review board at the Institute of Psychology, University of Wroclaw (#YBKOC/2021). Children provided assent to participate in the study, and informed written consent to participate was obtained in writing from their parent or their legal guardian.

### Participants

Using power analysis with G*Power (Faul et al., 2007), we estimated that a minimum of 54 participants is required to detect a moderate effect size of f = 0.25 (Mahmut et al., 2021a, b) with an alpha level of 0.05 and a power of 0.95. The sample invited to the study comprised 130 children aged between 6 and 10 years (54 children in the placebo group and 76 children in the experimental group), who were later assigned to either the experimental or the placebo group. The participants were recruited in public primary schools in Poland. The research team disseminated information about the project via municipality structures overseeing education. Information was sent to the teachers and headmasters, offering participation in scientific study during the “Science Day” at the University. We have also announced the project to the individual parents via the snowball method.

The study involved two testing sessions – a pre- and a post-training assessment. Despite numerous contact attempts from the research team, 10 children from the placebo group and 15 children from the experimental group did not come to the second testing session, and their data were not included in the analyses. Since compliance is important for olfactory training effectiveness (Pieniak et al., 2022), at the second testing session, parents reported training compliance of their child and additionally children self-reported their compliance. We decided to exclude from further analyses the data on 4 children from the experimental group whose compliance with the training regime was lower than 25%, as assessed by the parent or the child. By adopting such a liberal criterion, we assumed that in healthy children, when OT is not aimed at regenerating the olfactory system, metacognitive effects may appear with regular but less frequent exposure to odors. In the placebo group, the participants were not excluded based on the compliance report.

Prior to the testing sessions the parents or legal guardians were interviewed regarding child’s most prevalent medical conditions that could affect the sense of smell, such as chronic rhinosinusitis and traumatic brain injury (Welge-Lüssen et al., 2013). Additionally, before each testing session, children were asked whether they suffer from any current health problem and if they feel well at the time of the testing. Any health issue or medication reported by children was recorded at T1 and at T2 and we further compared odor identification scores of children with health issues/using medication with children self-assessed as completely healthy using independent samples t-tests. At T1, 23 children declared having some type of health problem (mostly allergies or slight colds), and 8 reported using some medication. At T2, 22 reported some health issues (again, mostly allergies and colds), and 12 – using medication. The odor identification scores were found to be independent of health status at T1 (*p* =.13), current medication at T1 (*p* =.48), health status at T2 (*p =*.21) or medication at T2 (*p* =.91), therefore no participants were excluded on that basis.

The final sample of participants comprised 101 participants (52 girls), 92 of whom were recruited from 5 different public schools and 9 who were enlisted individually by their parents. The children were aged between 6 and 9 years (*M* = 7.62 ± 0.61: 1 six-year-old, 43 seven-year-olds, 51 eight-year-olds and 6 nine-year-olds); 57 children in the experimental group (26 girls) and 44 children in the placebo group (26 girls). The groups of children retained and not included in the final sample did not differ statistically from each other in terms of age, verbal fluency, odor awareness and odor identification skills measured during the first session, as assessed by means of independent samples t-tests (see supplementary Table S1).

### Procedure

After contacting the research team, the participants were invited to the laboratory for the first testing session. They were told that the procedure would involve two testing sessions and a simple olfactory task that would need to be completed in the meantime.

The initial testing session occurred before the training, and the second session was scheduled 12 weeks after. Each participant underwent individual testing in a quiet, ventilated room. During each session participants completed questionnaires on odor awareness and significance, a verbal fluency test, and an olfactory identification test in a randomized order. Following the first session, participants were randomly assigned to either the experimental or the placebo group. Each person received a corresponding training set and detailed instructions. Training kits comprised four felt-tip pen odor dispensers (Sniffin’ Sticks, Burghart Messtechnik GmbH, Holm, Germany). The experimental group engaged in training with Sniffin’ Sticks containing four specific odors: eucalyptus (eucalyptol), cloves (eugenol), rose (phenyl ethyl alcohol), and lemon (citronellal) (Hummel et al., 2009). In parallel, the placebo group followed the same training routine using identical four pens but filled only with odorless propylene glycol. Participants, along with their parents or legal guardians, were instructed to smell each pen for 30 s, moving it from one nostril to the other, twice a day for 12 weeks. Training sessions were recommended to be conducted before meals or at least 30 min after eating.

Throughout the training, the children in both groups were using a custom-made compliance poster. Each child received a poster with 180 slots for the stickers and stickers were given to the parents/teachers. For each completed olfactory training session (90 days x 2 = 180 stickers) a child could stick one piece of the puzzle to the poster. When the training period was over, the poster revealed a collage of images of smelling items. Data on training compliance were collected during the second appointment from both the children and their guardians. Data were collected between Winter 2021 and Autumn 2022 (9 months in total).

## Materials

### Odor significance and awareness

The main focus of the current study was odor significance and awareness in young children. Among metacognitive measures available for the youngest age groups, the most popular tool focused on children’s olfactory perception of the surrounding world is the “Children’s Olfactory Behaviors in Everyday Life” (COBEL) scale – a comprehensive, 16-item self-report questionnaire developed by Camille Ferdenzi and colleagues (Ferdenzi et al., 2008a, b). This semi-qualitative scale focuses on children’s olfactory perceptions and behaviors in three domains: social, environment- and food-related. Since we wanted to compare odor awareness and significance before and after olfactory training in young children, we wanted to modify this scale to make it (a) understandable for children; (b) self-reported; (c) balanced between food, environment, and social domains; (d) appropriate in the culture of the participating children; (e) short enough even for young children with a short attention span; and (f) quantitative, so that the scores could be easily comparable between children (see also Sorokowska et al., 2024).

The final version of the scale had 10 items (see supplementary File S2 for a detailed description of the scale modification), with scores ranging between 0 and 2 for each item. For further analyses, a total score was computed for each child (range 0–20). Consistent with reports on questionnaire self-assessment quality in young children (de Leuuw & Otter, 1995), the reliability of the final version of the scale was moderate but satisfactory (T1: α = 0.57; T2: α = 0.58). It should be noted here that the responses in item 1 on “odor in food dislikes” were highly skewed (most participants scored 0), suggesting that this item might be difficult for young children.

### Olfactory identification

Odor identification skills were tested with U-Sniff test (Schriever et al., 2018a, b): a standardized, medical olfactory test designed for children and adapted for use in many countries, including Poland, where the current study was conducted. Odor identification ability is assessed in a forced-choice task involving 12 common odorants. A child is asked to choose a name of a presented odor from a list of four of alternatives and gets one point for each correct identification, with final scores ranging between 0 and 12.

### Verbal fluency

To explore other, potential effects of olfactory training on the functioning of young children, we also tested verbal fluency of our participants. The verbal fluency test is a short measure developed by Benton and colleagues (Benton et al., 1983). In this simple task, participants are asked to come up with as many words beginning with a certain letter as they can. This needs to be done within one minute per letter, and the typical letters included in this task are F, A and S.

### Data analysis

Our analyses focused on three dependent variables: odor significance and awareness, odor identification skills, and verbal fluency. We have first explored the inter-correlations of all dependent variables at T1 and T2 by means of Pearson’s *r* correlations. The main analysis comprised separate, repeated measures ANCOVAs with a within-subject effect of training (T1 before vs. T2 after the training), and a between-subject effect of group (0 placebo vs. 1 experimental). The children in the placebo group were slightly older than the children in the experimental group (7.91 ± 0.60 vs. 7.39 ± 0.53, *p* <.001), therefore age was controlled in the statistical analyses. Boys and girls reported similar odor significance and awareness, *F*(1,98) = 1.54; *p* =.22, while controlling for age, and odor identification skills, *F*(1,98) = 1.86; *p* =.18, while controlling for age. However, girls scored higher than boys in the verbal fluency test, *F*(1,98) = 4.10; *p* =.045, with a mean difference: 1.2. We decided against including gender in the subsequent analyses to retain sufficient statistical power.

## Results

### Olfactory training effects

All descriptive statistics for the main variables measured at T1 and T2 in the experimental and placebo groups are presented in Table 1.

**Table 1:** Odor significance and awareness, odor identification, and verbal fluency scores in the experimental and placebo groups as assessed during the first and second testing sessions

	Odor significance and awareness [0–20]				Odor identification [0–12]				Verbal fluency [0+]			
	T1		T2		T1		T2		T1		T2	
	Pla	Exp	Pla	Exp	Pla	Exp	Pla	Exp	Pla	Exp	Pla	Exp
M	10.16	9.07	10.7	9.65	10.25	9.79	10.07	9.91	6.59	5.19	6.50	5.61
SD	2.77	2.93	2.88	2.86	1.38	1.75	1.39	1.47	2.97	3.14	2.44	2.88
SE	0.42	0.39	0.46	0.38	0.21	0.23	0.21	0.19	0.45	0.42	0.37	0.37
Min	4	4	4	4	7	4	5	5	0	0	2	0
Max	15	16	17	16	12	12	12	12	15	16	13	14

Note: T1-first testing session; T2-second testing session; Pla-Placebo group; Exp-Experimental group

Odor significance and awareness at T1 significantly correlated with verbal fluency (*r* =.31, *p* =.002) and with odor identification skills (*r* =.31, *p* =.002). Verbal fluency at T1 also correlated with the olfactory identification score (*r* =.23, *p* =.02). Odor significance and awareness at T2 significantly correlated with verbal fluency (*r* =.27, *p* =.006), but not odor identification (*r* =.07, *p* =.48). Odor identification and verbal fluency were not correlated at T2 (*r* =.01, *p* =.92).

### Effects of OT on odor significance and awareness

**Fig. 1:**
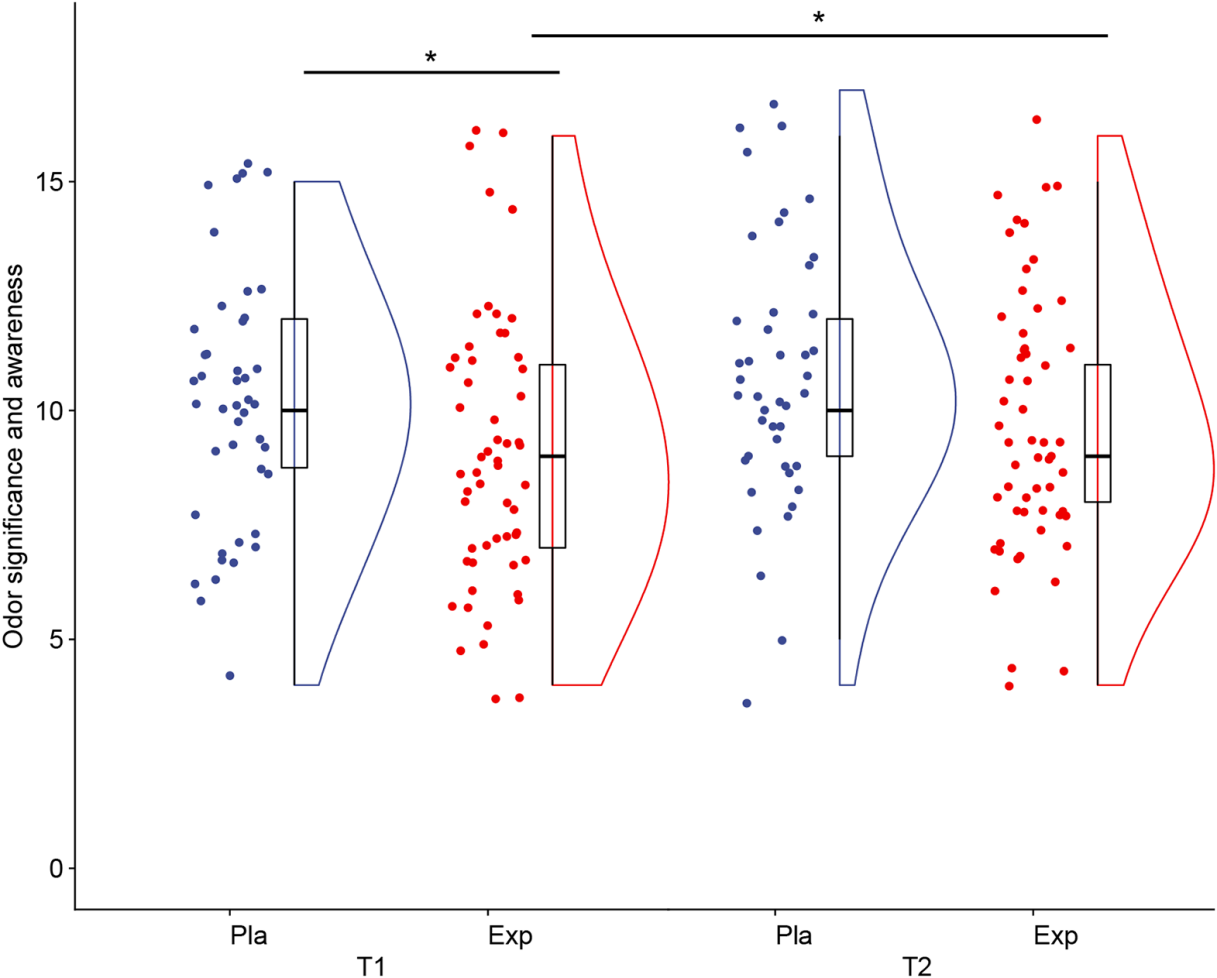
Individual scores at both testing sessions for experimental and placebo groups. *Note*: T1 – first testing session; T2 – second testing session; Pla-Placebo group; Exp-Experimental group

We noted a significant main effect of group, *F*(1,98) = 5.2, *p* =.03, η^2^ = 0.05, with the experimental group tending to score 1.25 point lower than the placebo group across the two testing sessions. The main effect of training was on a trend level, *F*(1,98) = 3.65, *p* =.06, η^2^ = 0.04, suggesting a marginal improvement in both groups. The interaction effect of training and group was non-significant *F*(1,98) = 0.82, *p* =.37, η^2^ = 0.04, however, the post-hoc analysis of simple effects revealed that the experimental group declared lower odor significance and importance at baseline than the placebo group (*p* =.02) and that between the testing sessions, the experimental group odor significance and awareness increased (*p* =.04), while no such change was noted in the placebo group (*p* =.59), leading to the non-significant between-group difference in odor significance and awareness at the follow-up measurement (*p* =.13) (See Fig. 1). Training and age interaction was significant, *F*(1,98) = 4.24, *p* =.042, η^2^ = 0.04 pointing to the slightly more pronounced increase in odor awareness in older children (*r* =.18, *p* =.07). For all descriptive statistics see Table 1.

### Odor identification

The olfactory training had no significant effect on odor identification skills in either of the study groups [non-significant interaction training*group, *F*(1,98) = 1.72, *p* =.19, η^2^ = 0.02; non-significant main effect of training, *F*(1,98) = 1.80, *p* =.18, η^2^ = 0.02; non-significant main effect of group, *F*(1,98) = 0.52, *p* =.47, η^2^ = 0.005]. Odor identification score remained on a level similar to baseline performance following the olfactory training in both groups (all *p*s > 0.18). The age covariate also proved to be non-significant, *F*(1,98) = 4.24, *p* =.042, η^2^ = 0.04, and did not interact with the olfactory training, *F*(1,98) = 1.1, *p* =.30, η^2^ = 0.01. For all descriptive statistics see Table 1.

### Verbal fluency

The olfactory training had no significant effect on the verbal fluency score – the performance of placebo and experimental groups remained virtually unchanged following the olfactory training [non-significant interaction training*group, *F*(1,98) = 0.98, *p* =.33, η^2^ = 0.002; non-significant main effect of training, *F*(1,98) = 0.242, *p* =.624, η^2^ < 0.001; non-significant main effect of group, *F*(1,98) = 1.91, *p* =.17, η^2^ = 0.008] (all *p*s > 0.32). Age was a marginally significant covariate *F*(1,98) = 3.24, *p* =.075, η^2^ = 0.03, suggesting higher overall verbal fluency (mean score from both measurements) in older children (*r* =.25, *p* =.01). For all descriptive statistics see Table 1.

## Discussion

People differ in the attention they pay to olfaction in their daily lives and the awareness of olfactory cues can change and develop through different experiences. At the same time, olfactory training can significantly affect both olfactory and cognitive functioning. Here, we sought to experimentally test whether odor significance and awareness in young children can be developed through standardized olfactory training. Additionally, we explored whether such training can improve odor identification skills and verbal fluency in young children. The intervention was found to marginally affect the development of odor significance and awareness. Further, olfactory training affected neither the odor identification nor the verbal fluency scores in our sample.

The OT method we applied in this study was advanced with a novel addition of the placebo group. The strict control design was meant to allow for a potential disentangling of the cognitive effects of odorous stimulation from the consequences of repeated, structured odorless sniffing and daily focus on an olfactory behavior. Cross-sectional (Martinec Nováková & Vojtušová Mrzílková, 2016) and longitudinal works (Martinec Nováková et al., 2018) suggest that the olfactory diversity of children’s environments predicts an increased odor awareness. In our study, the olfactory significance and awareness score improved in the experimental group. Regular act of smelling actual odors and an intense focus on the olfactory sensation seems to have generated a slight increase in olfactory metacognition. It is important to note that the increase in olfactory metacognition was noted for an experimental group that had lower scores at baseline than the placebo group. Although unintentional, this difference limits our conclusions about the effectiveness of olfactory training to individuals with lowered baseline olfactory metacognition. Consequently, the clinical populations characterized by lower attention to odors should be considered as a target group for follow-up studies. Children who have difficulties in conscious perception of daily odors, such as children after traumatic brain injury (Pieniak et al., 2023) or with autism spectrum disorder (Schriever et al., 2018a, b), would likely benefit from olfactory training in a more conclusive way than the healthy population.

The finding that olfactory training can enhance olfactory metacognition is convergent with other preliminary evidence coming from the adult population showing that in some cases, mindfulness training combined with mindful perception of environmental odors may enhance olfactory metacognition (Mahmut et al., 2021a, b). Together, these studies suggest that metacognitive aspects of olfactory perception can be induced with easy, enjoyable daily activities focused on odors. However, a recent study demonstrated that six-weeks, regular after-school workshops engaging the sense of smell do not increase olfactory abilities or the importance of the sense of smell in children aged 9–11 years (Martinec-Novakova & Markova, 2025). Concluding the potential source of divergent findings between our study and the one reported by Martinec-Novakova & Markova is hindered by the different ages of the participants (6–9 vs. 9–11 years old). Thus, given the preliminary nature of our study and still fragmentary knowledge about the effectiveness of olfactory training in children, we acknowledge that additional data is required to draw definitive conclusions. Ideally, the intervention we implemented should also be compared to alternative methods aimed at enhancing children’s olfactory focus, such as structured smell workshops and extended classes on olfaction.

Despite careful planning, the drop-out and compliance issues have significantly decreased the number of children who underwent the second testing session. The dropout, however, seems to be random, not driven by a certain pattern of results obtained in the tests that children took at the baseline. We therefore recommend that this preliminary trend we report is followed up by more extensive research, preferably in larger groups of more diverse age range. Should the olfactory significance and awareness increase following OT be reconfirmed, promising learning and developmental conclusions could be reached. This pleasant, enjoyable, and harmless method could easily become an additional element of daily school routine as a support method for arousing olfactory significance and awareness. Provided the potential emotional and behavioral correlates of odor awareness (Croy et al., 2010; Martinec Nováková & Havlíček, 2019), it may be assumed that such outcome may further translate to a much broader scope of effects, exceeding those pertaining purely to the olfactory perception.

Following the research on olfactory system plasticity and its close associations with various brain structures, we explored the potential effect of olfactory training on verbal fluency and odor identification skills. Despite some effect olfactory training had on the odor significance and awareness, we found that verbal fluency remained on a similar level at T1 and T2 in both the experimental and the placebo group. This finding is not consistent with previous reports on adults [elderly people (Birte-Antina et al., 2018), normosmic and dysosmic adults (Oleszkiewicz et al., 2022)]. However, provided the strong association of verbal fluency with age (observed also in our sample) this finding might mean that OT is not strong enough as to overpower the prevailing developmental dynamics in verbal development of healthy children (Oleszkiewicz et al., 2016). Nevertheless, positive outcomes of olfactory training were previously reported for fluid intelligence in children with traumatic brain injury (Pieniak et al., 2023), thus the subtle differences between cognitive abilities which can be augmented by olfactory training, and which remain unchanged following this intervention, definitely warrant further investigation. Particularly important are further scientific explorations of OT utility in children with cognitive deficits. It would be also very interesting to observe whether other aspects of meta-perception or self-assessments of one’s cognitive and olfactory skills would increase as a result of OT.

It should be noted that we did not observe any increase in odor identification skills following the olfactory training. The lack of such an effect in our sample adds another data point to the inconsistent picture of effects olfactory training has on odor identification skills in healthy young children [see (Pieniak et al., 2023) vs. (Mahmut et al., 2021a, b)]. It may be presumed that at least some of these inconsistencies relate to an overall, high performance of healthy children in the U-Sniff test (Schriever et al., 2018a, b) that was primary designed as a screening tool for olfactory dysfunctions. The meta-analysis of previous findings on olfactory training effectiveness has indeed shown a considerable effect it has on odor identification abilities, but the studies included in that analysis were conducted in clinical samples (Sorokowska et al., 2017). Perhaps, the sensitivity of the U-Sniff test may be insufficient to detect subtle increases in olfactory skills in children of no preexisting olfactory deficits. This issue could be further addressed in extended studies combining various methods of olfactory testing. For example, free identification is definitely a more difficult task than cued identification, and in addition to olfactory abilities it also involves a considerable cognitive effort. Building upon alterations in functional connectivity the OT may drive in normosmic participants (Al Aïn et al., 2019), it could be presumed that such a task, difficult both at an olfactory and at a cognitive level, would be more likely to demonstrate olfactory training-induced changes. Another arising question is whether olfactory metacognition mediates the effects of olfactory training on olfactory abilities. Considering the rather small effects found in this study, and the null effects of OT on odor identification, this problem should require intense experimental efforts involving larger study samples and more sensitive measures of olfactory abilities.

Our study does not test for the potential differences between schools or classes that participated in our study. One school may present better quality of teaching or simply gather children from families with higher socioeconomic status than another school. Nevertheless, the schools included in this project were all public and located in roughly similar neighborhoods. Further, one class may benefit from a more enthusiastic teacher than the other. Another complication is the fact that across all classes included in the project 6–18 children were tested twice. A typical school class includes approximately 25–30 children and we cannot tell if attending both testing sessions (or dropping out) within each class was random or not. Lastly, our a priori sample size estimation did not take the school/class parameter into account. To avoid the inflation of type II error risk, we decided against statistical testing of this potential confound. Future attempts should consider variation grouped in schools/classes when investigating metacognitive aspects of olfaction as a derivative of cognitive and socioeconomic background.

Based on the current investigation and the previous literature we conclude that OT might potentially have a small effect on olfactory metacognition in children with low olfactory performance and olfactory awareness at baseline. Future studies should explore the effects of olfactory training on olfactory metacognition in children from clinical groups characterized with olfactory deficits and difficulties in conscious perception of odors, such as children after traumatic brain injury or autism spectrum disorder.

## Electronic supplementary material

Below is the link to the electronic supplementary material.

Supplementary Material 1

## Data Availability

The data underlying this article will be shared upon request to the corresponding author.
