# Supplementary material for: Olfactory training and metacognitive aspects of olfaction in children aged 6–9 years: a preliminary study
Source: Psychol Res. 2025 May 20;89(3):100. doi: 10.1007/s00426-025-02127-y (PMC12092523; doi:10.1007/s00426-025-02127-y)
Supplement: Supplementary file 1 — Supplementary Material 1 [file 426_2025_2127_MOESM1_ESM.docx]

**Supplementary materials**

**Table S1.**

Independent samples t-tests: differences between children retained and not included in the final sample.

|  | **Group** | **Mean** | **SD** | **SEM** | **t** | **p** | **Effect Size (Cohen's d)** |
| --- | --- | --- | --- | --- | --- | --- | --- |
| Age | 0 | 7.74 | 0.83 | 0.15 | 0.84 | 0.402 | 0.18 |
|  | 1 | 7.62 | 0.61 | 0.06 |  |  |  |
| Verbal fluency | 0 | 5.97 | 4.28 | 0.80 | 0.23 | 0.821 | 0.05 |
|  | 1 | 5.80 | 3.13 | 0.31 |  |  |  |
| OSA total score | 0 | 9.61 | 2.99 | 0.56 | 0.10 | 0.920 | 0.02 |
|  | 1 | 9.54 | 2.90 | 0.29 |  |  |  |
| Odor identification score | 0 | 9.83 | 2.32 | 0.43 | -0.43 | 0.667 | -0.09 |
|  | 1 | 9.99 | 1.61 | 0.16 |  |  |  |

*Note: Group 1 – scores of 101 children retained in the sample; 0 – 29 children not included in the final sample SEM - Standard error of the mean, OSA – Odor Significance and Awareness*

**Supplementary file S2**

Description of the COBEL scale modifications.

Since we wanted to compare odor awareness and significance before and after olfactory training in young children, we wanted to modify this scale to make it (a) understandable for children and applicable as a self-report, (b) quantitative, so that the scores could be easily comparable between children, (c) balanced between food, environment, and social domains, (d) appropriate in the culture of the participating children, (e) short enough even for young children with a short attention span.

To this aim, we first decided to remove some items from the “environment” component (3,6,8,9,10,15) and we split the original COBEL “food” item number 2 in two (“Imagine you see a dish you do not know: will you smell it before putting it in your mouth (Often / sometimes / never)?” and “I smell on food before tasting it” (Often / sometimes / never)). This step resulted in obtaining 4 items per food, environment, and social domains. Elimination of some items also helped simplify the scale [e.g., in Novakova et al (2019) study, the authors also removed the question on olfactory sensations in nature, as it was difficult for many participants]. Further, we simplified three complex items (numbers 1,13 and 14 in the original COBEL scale). For example, the original item 1. on odor in food dislikes read: “Are there some foods/drinks that you hate (yes/no)? Which ones (up to 6)? For which reasons (for each cited food)?”. In our study we used a simpler version that read “Are there some foods/drinks that you hate because of their smell? Which ones (up to 6)?”).

The preliminary version of our odor significance and awareness scale contained complex and abstract items on “Outside odors” and “Family odors” from COBEL (Ferdenzi, Mustonen, et al., 2008), wherein the participants were asked to imagine there would be no outside/family member odors anymore and assess how they would feel in this case. However, these questions proved to be very difficult to understand for some participants. This was probably related to the abstract thinking skills necessary to understand the meaning of these questions. Further, discussion within the research team revealed that response options “would you not care/would it bother you/would it suit you” could not be coded and quantified easily (the team did not agree on whether being bothered by odors pertained to high or low odor significance and awareness). Therefore, despite being included in the initial stage of the project, we have further decided not to continue using these items in the final version of the scale.
